# Supplementary material for: FIAEPI-KD: A novel knowledge distillation approach for precise detection of missing insulators in transmission lines
Source: PLoS One. 2025 May 30;20(5):e0324524. doi: 10.1371/journal.pone.0324524 (PMC12124544; doi:10.1371/journal.pone.0324524)
Supplement: Attachment — Submitted filename: comments - Insulator-detection.docx [file pone.0324524.s001.docx]

This work looks pretty good. I request editor to give major revision regarding this work. The following comments I suggest in this work are.

1. Rewrite the abstract. It is not looking fine.
2. Add/ modify one more contribution of your network.
3. Provide a visual comparison of various state of the art networks on the used datasets in this work.
4. We may show comparison of computational complexity analysis in terms of parameters, FLOPS, MAC and runtime.
5. Include the most recent related works : <https://doi.org/10.32604/cmc.2024.051844> , <https://doi.org/10.1007/978-981-99-0189-0_4> , <https://doi.org/10.1007/s00034-025-03009-9>, <https://doi.org/10.1080/02564602.2023.2176932>, <https://doi.org/10.1007/s00034-024-02837-5>
6. Rectify spacing and typo errors in this paper.
7. Discuss few limitations of this work and future work in the last section.
